# Gap geometry dictates epithelial closure efficiency

**DOI:** 10.1038/ncomms8683

**Published:** 2015-07-09

**Authors:** Andrea Ravasio, Ibrahim Cheddadi, Tianchi Chen, Telmo Pereira, Hui Ting Ong, Cristina Bertocchi, Agusti Brugues, Antonio Jacinto, Alexandre J. Kabla, Yusuke Toyama, Xavier Trepat, Nir Gov, Luís Neves de Almeida, Benoit Ladoux

**Affiliations:** 1 Mechanobiology Institute, National University of Singapore, Singapore 117411, Singapore; 2 Sorbonne Universités, UPMC University Paris 06, CNRS UMR 7598, Laboratoire Jacques-Louis Lions, F-75252 Paris, France; 3 CEDOC - Chronic Diseases Research Center, NOVA Medical School, Rua Camara Pestana, 6, Lisbon, 1150-082 Lisbon, Portugal; 4 ICREA at Institute for Bioengineering of Catalonia and Universitat de Barcelona, 08028 Barcelona, Spain; 5 Department of Engineering, University of Cambridge, Cambridge CB2 1PZ, UK; 6 Department of Biological Sciences National University of Singapore, Singapore 117543, Singapore; 7 Temasek Life Sciences Laboratory, Singapore 117604, Singapore; 8 Weizmann Institute of Science, Rehovot 76100, Israel; 9 INRIA-Paris-Rocquencourt, MAMBA Team, 78153 Le Chesnay, Domaine de Voluceau BP105, France; 10 Institut Jacques Monod (IJM), CNRS UMR 7592 and Université Paris Diderot, 75013 Paris, France

## Abstract

Closure of wounds and gaps in tissues is fundamental for the correct development and physiology of multicellular organisms and, when misregulated, may lead to inflammation and tumorigenesis. To re-establish tissue integrity, epithelial cells exhibit coordinated motion into the void by active crawling on the substrate and by constricting a supracellular actomyosin cable. Coexistence of these two mechanisms strongly depends on the environment. However, the nature of their coupling remains elusive because of the complexity of the overall process. Here we demonstrate that epithelial gap geometry in both *in vitro* and *in vivo* regulates these collective mechanisms. In addition, the mechanical coupling between actomyosin cable contraction and cell crawling acts as a large-scale regulator to control the dynamics of gap closure. Finally, our computational modelling clarifies the respective roles of the two mechanisms during this process, providing a robust and universal mechanism to explain how epithelial tissues restore their integrity.

## Abstract (web summary)

Epithelial wound closure proceeds through both crawling into the wound and by constricting an actomyosin cable in a so-called purse-string mechanism. Here the authors show that the two mechanisms are mechanically coupled and the curvature of the wound regulates the overall dynamics of wound closure.

## Graphical abstract

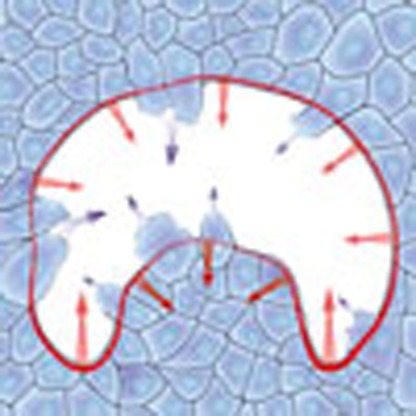

Gaps in multicellular tissue naturally occur during the lifetime of an organism, from developmental stages to adult life. Holes within tissues may be natural consequences of physiological processes such as development, organ remodelling and extrusion of apoptotic cells^1^,^2^,^3^,^4^. Alternatively, they may be the outcome of pathological or injurious events and result in ulcers and wounds^5^,^6^. In both cases, gaps need to be promptly closed to re-establish the physiological functions of the tissue^7^. To this end, cells surrounding a void engage in coordinated movement in order to close gaps and wounds using two main distinct mechanisms: crawling of cells on the substratum and constriction of a supracellular actomyosin cable at the edge of the gap in a purse-string-like mechanism^4^,^6^,^8^,^9^,^10^,^11^,^12^,^13^,^14^,^15^. In the presence of the extracellular matrix (ECM), cell crawling with lamellipodia protrusion^16^,^17^ seems to be the predominant mechanism used to close the voided area, particularly when the gap is a large one. Owing to the stochastic nature of lamellipodia formation, this closure mechanism is characterized by a ‘rough' edge^8^. Often it has been observed that one cell takes the lead and drags the neighbouring cells along^17^. On the other hand, purse-string closure involves the coordinated movement of all the cells at the edge due to ATP-dependent constriction of the actomyosin cable. Supracellular coordination of this motion induces the shape of the gap to become rounder and with ‘smooth' edges^1^,^18^,^19^. This mechanism has been observed to be involved in closure of small gaps such as those left behind by apoptotic cells^4^,^20^ and in closure of gaps in epithelia with poorly developed ECM, as in the case of embryonic epithelia and corneal epithelium in adults^21^,^22^. However, the two mechanisms are not mutually exclusive and, depending on the biochemical and biophysical properties of the environment, they may contribute to different extents to the closure of the void^19^,^21^,^22^,^23^,^24^,^25^,^26^. A number of factors have been proposed to regulate the closure mechanisms including ECM coating, cell type, substrate stiffness and gap size^9^,^10^,^27^,^28^. These factors may contribute to alter the roughness of the edge leading to the coexistence of large lamellipodia formation together with the formation of discontinuous contractile actin cables^9^. At the cell–substrate interface, our recent study showed that not only do lamellipodia extensions towards the gap contribute to epithelial closure, but also the compression of the underlying substrate by discontinuous contractile actin cables connected to focal adhesions promotes efficient wound closure^29^.

The intrinsic complexity of this picture has fuelled a long-lasting debate on the relative contribution of crawling and purse-string to re-establish integrity of the epithelium. Indeed, the variability of the experimental settings used to create the voids—damage versus damage-free gaps, *in vitro* versus *in vivo* experiments—and the environmental conditions within the gap, for example, presence of ECM, are likely to lead to only partial conclusions. In this work, we used microfabrication techniques to create damage-free gaps of geometries containing both convex and concave features. This allowed us to determine the importance of tissue shape in epithelial gap closure and to decipher the role of curvature in this process. An essential difference between the two closure mechanisms is that cell crawling always pulls the edge of the tissue forward (that is, towards the gap) while purse string pulls the edge forward or backwards depending on the local geometry. Our study demonstrates how the interplay between these two mechanisms is crucial for closing gaps and wounds, which naturally come in arbitrary shapes.

## Results

### Closure of gaps of varying geometries

**Figure 1:**
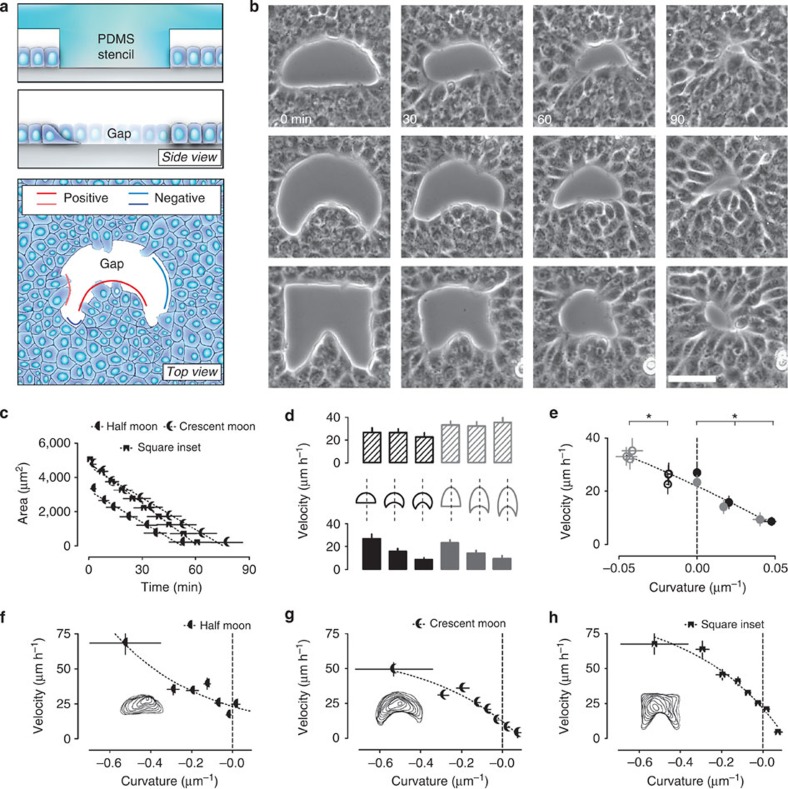
Closure of gaps and wounds of different geometries is curvature-dependent. (**a**) Cartoons showing *in vitro* generation of damage-free gaps in an epithelial wound model experiment. Top, a PDMS stencil serves as a block to create damage-free gaps with well-defined geometries. Middle, after removal of the stencil, epithelial cells move into the voided area. Bottom, top view of a gap after removal of the stencil. In this paper, the epithelium protruding into the gap (where the tissue is locally convex) is defined as having a positive curvature. Conversely, concave regions are defined as being negatively curved. (**b**) Example time lapses of gap closure. Voids of varying geometries were created in confluent and mature epithelia. Scale bar, 50 μm. (**c**) Decay in the area of differently shaped gaps over time (*n*=15–18). (**d**) The cartoon shows the six different moon-like gaps used to test dependence of velocity on local curvature. The edge movement velocity is measured at the intersection between dashed line and the north (negative curvature) or south pole (flat or positive) of the gap. Bar plots display the edge movement velocity measured at the intersection between the dashed line and the north (negative curvature=textured bars) or south pole (flat or positive curvature=full bars) of the gap. (**e**) Plot of velocity as a function of curvature from the experiments shown in **d**. Empty and full circles correspond to top and bottom bar plots in **d**, respectively. The dashed line indicates linear fitting of data as a visual guide. Stars indicate a statistically significant difference of velocity (*P*<0.05; *n*=12–23). (**f**–**h**) Velocity plots of randomly chosen points at the edge of gaps as in **b**. Logarithmic fitting (best fit; *R*^2^=0.89–0.98) of experimental data are shown by dashed line as a visual guide (*n*=15–18). Insets, overlay of outlines at different time points from representative experiments. Error bars indicate s.e.m. Samples are considered statistically different for *P*<0.05 in unpaired Student's *t*-test and are indicated by the star.

Undamaged gaps with different initial shapes were formed in confluent epithelia by growing MDCK (Madin-Darby canine kidney) cells for 12–18 h around polydimethylsiloxane (PDMS) stencils of well-defined geometries (Fig. 1a and Supplementary Fig. 1). Closure of gaps started immediately after removal of the PDMS block (Fig. 1b) and the area of the gap decreased in a near-linear manner irrespective of the initial shape (Fig. 1c). Since poorly developed ECM modifies cell–substrate adhesion, hindering crawling and favouring actin bundle assembly, we tested different concentrations of fibronectin. Consistent with previous findings showing increased migration of single cells with higher amounts of ECM on the substrate^30^, we found that collective migration of MDCK cells in a wound model experiment was increased by incremental amounts of fibronectin (Supplementary Fig. 2). Importantly, the response to fibronectin dosage was sigmoidal with the half maximal effective concentration (EC50) observed at ∼20 μg ml^−1^ loading concentration. We chose this concentration to perform our gap-closure experiments.

Despite these common features, local regulation of the closure velocity was evident from our experiments: concave edges (protruding into the gap)—that is, positively curved edges—clearly advanced at a slower pace compared with convex—that is, negatively curved—or flat edges (Fig. 1b and Supplementary Movie 1). Furthermore, prominent positive regions remained on halt until neighbouring negative regions reached up to the same level and only then did the edge advance (recalling theoretical study^31^). To systematically verify these observations, we combined semicircles and semiellipses to produce moon-shaped gaps of increasing curvatures, both positive and negative (Fig. 1d). The higher the negative curvature, the faster the edge moved (Fig. 1d, top). Conversely, the higher the positive curvature, the slower the movement (Fig. 1d, bottom). Our data showed that the local curvature was a major regulator of the gap edge velocity for all shapes tested, with velocity decreasing with increasing curvature (Fig. 1d–h).

### Local regulation of crawling and purse-string mechanisms

**Figure 2:**
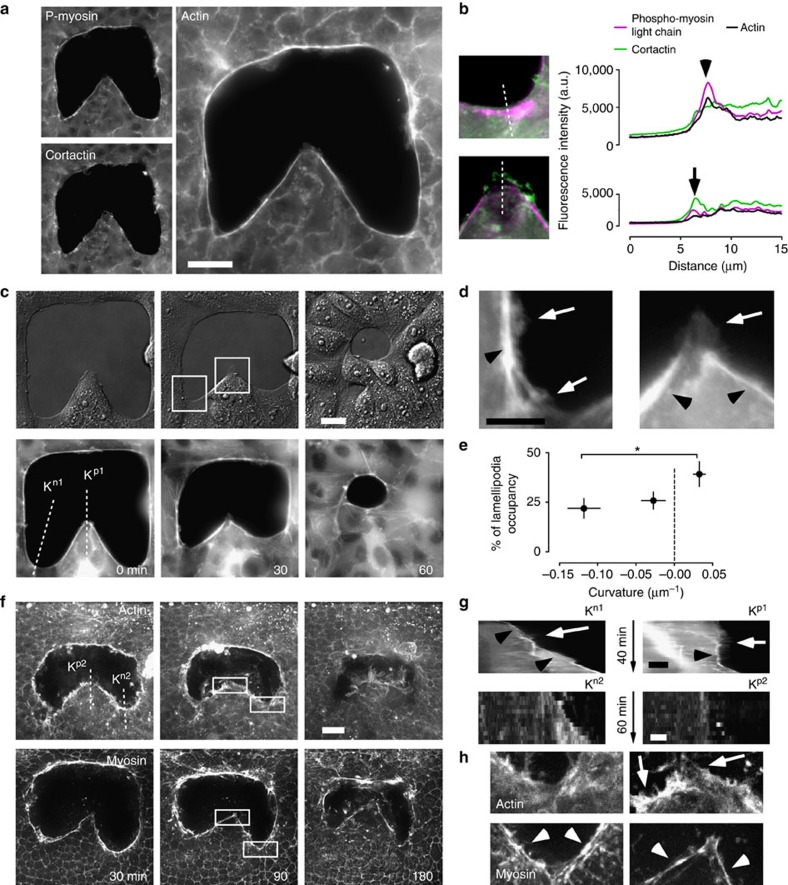
Crawling and purse-string show curvature dependence. (**a**) Immunofluorescence for cortactin, phosphomyosin light chain and actin localization 15 min after stencil removal. Staining of phoshorylated myosin light chain (top left) and cortactin (bottom left) and actin (right). Scale bar, 20 μm. (**b**) Left, enlarged view of negative and positive regions (grey, actin; green, cortactin and magenta, phosphomyosin). Right, fluorescence intensity line profiles of the dashed lines. (**c**) Time lapse of gap closure visualized by DIC (top) and GFP-Actin in stably transfected MDCK cells (bottom). GFP-Actin levels vary between cells, and thus cannot be used for quantitative comparison. K^p1^ and K^n1^ are lines used to generate kymographs in **g**. Scale bar, 20 μm. (**d**) Enlarged view of boxes in **c**. Scale bar, 10 μm. (**e**) Percentage of lamellipodia occupancy of the edge as a function of the local curvature. The gap edge is subdivided into three regions according to their curvature. Length of the edge occupied by lamellipodia is expressed as a percentage of the total length of that particular region. Measurements are taken from still images 15 min after the removal of the stencil (*P*<0.05; *n*=63 independent regions from 17 experiments). (**f**) Examples of *in vivo* wound closure with varying geometries. Wounds were made with laser microsurgery on notum epithelia of *D. melanogaster* pupa expressing mCherry-Moesin as a marker for actin (top) and Spaghetti-Squash-GFP as a myosin marker (bottom). K^p2^ and K^n2^ are lines used to generate kymographs in **g**. Scale bar, 50 μm. (**g**) Kymographs from the lines in **c**,**f**. Lamellipodia protrusion at positive regions were more persistent then those at negative once. Scale bar for top panels, 10 μm; for bottom panels, 5 μm. (**h**) Enlarged view of boxes in **f**. In all images, arrowheads indicate the actomyosin cable and arrows indicate lamellipodia. Error bars indicate for s.e.m. Samples are considered statistically different for *P*<0.05 in unpaired Student's *t*-test and are indicated by the star.

Crawling and purse-string, the two major mechanisms contributing to gap closure, are both promoted by actin structures. Indeed, immunofluorescence staining for actin cytoskeleton, lamellipodia (cortactin) and purse-string (phosphomyosin light chain) revealed the presence of both structures at the free edge of the epithelium (Fig. 2a). An actomyosin cable of varying intensity could be detected in all cells surrounding the gap with the highest intensity of both actin and phosphomyosin detected at negative curvatures (Fig. 2b, top). Importantly, the cable structure was continuous between adjacent negative and positive regions, demonstrating that they are mechanically connected. Lamellipodia also appeared to be distributed along the whole edge, with a preference for the tip of the positive regions^32^ where the cable appeared to be weaker (Fig. 2b, bottom). This result is in agreement with recent findings showing that weakening of the cable is a stimulus for lamellipodia formation^17^. To further investigate the universality of the relationship between actin organization and tissue curvature, we analysed the distribution of actomyosin cables and lamellipodia in a model wound assay^10^,^33^. Immunofluorescence for actin structures in spontaneously formed curvatures revealed conserved features as in our gap-closure experiments with purse-sting predominant in negative curvatures and lamellipodia in positive curvatures (Supplementary Fig. 3). MDCK cells stably transfected with GFP (green fluorescent protein)–Actin were used to investigate how closure dynamics at different curvatures related to crawling and purse-string mechanisms (Fig. 2c and Supplementary Movie 2). Clear morphological variation as well as differences in cell dynamics were noted: lamellipodia were visibly less pronounced and less persistent in fast-moving negative regions compared with slow-moving positive ones (Fig. 2d,g). This was confirmed by the analysis of lamellipodium distribution (Fig. 2e), which was significantly more pronounced in positive regions (21.9±5% lamellipodium edge occupancy for negative regions *n*=26, 39.2±6.5% for positive, *n*=16, mean±s.e.m., *P*<0.05 in unpaired Student's *t*-test). On the other hand, negative curvature regions exhibited prominent and uninterrupted actomyosin cables at the gap edge, whereas discontinuous cables at the back of the protrusive lamellipodia were observed at the tip of positive curvature regions. Taken together, these results showed that negative curvatures stabilized the formation of contractile actin bundles, which are not perturbed by the appearance of small and transient lamellipodia. Conversely, positive curvatures favoured the assembly of persistent lamellipodial extensions, which may perturb the continuity of the actomyosin cable in particular at the positive regions (Fig. 2d, right panel and Fig. 2g, top panels).

### *In vivo* closure of non-convex wounds

The relevance of our *in vitro* studies of epithelial gap closure was confirmed by the study of non-convex wounds on the notum epithelium of *Drosophila melanogaster* (Fig. 2f–h and Supplementary Movie 3). Interestingly, closure of *in vivo* wounds showed a similar dependence between edge movement and local curvature as observed *in vitro* (Fig. 2f,g), with wound closure starting and progressing faster at negatively curved regions than positive ones. Consistent with *in vitro* observations, positive curvatures promoted a crawling mechanism while negative curvatures favoured the appearance of thick actomyosin cables (Fig. 2h and Supplementary Fig. 4). Altogether, results obtained in these highly complementary systems suggest an underlying universal and conserved mechanism relating epithelial closure and tissue geometry.

### Focal adhesions and mechanical force distribution

**Figure 3:**
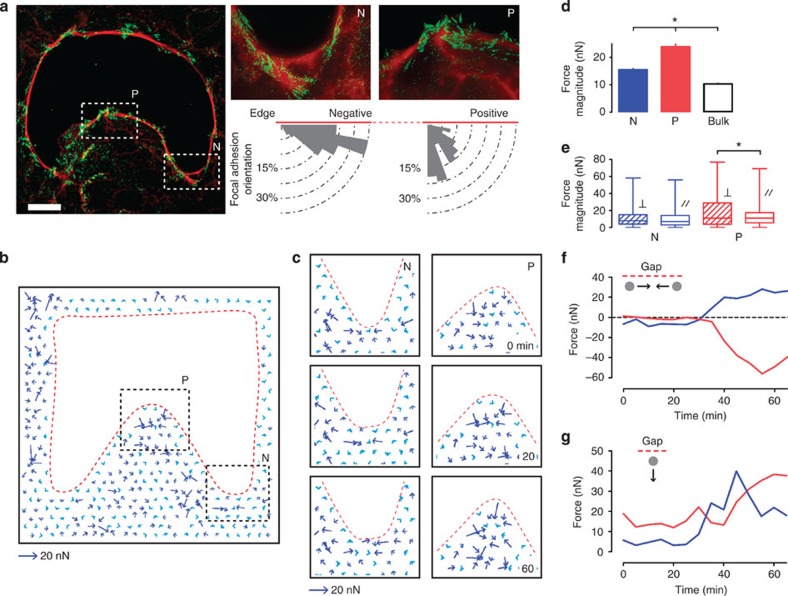
Focal adhesions and traction forces at the gap edge. (**a**) Left, immunofluorescence staining of actin (red) and paxillin (green) at 15 min after removal of the stencils show the distribution and orientation of focal adhesions at the gap edge. Top right, representative super resolved (SIM) images were used to quantify angular orientation of focal adhesions (bottom) at different curvatures (N, negative, P, positive). Data represent cumulative counts from four independent gaps. Scale bar, 20 μm. (**b**) Analysis of force distribution. Contrast of force vectors scales with magnitude (dark blue for forces >15 nN). (**c**) Enlarged view of dashed boxes in **b**, showing the evolution of force distribution over time. (**d**) Force magnitude plot in negatively (N) and positively (P) curved regions. Bulk shows the average force magnitude in regions >15 μm away from the edge. Force magnitudes are the average force per pillar in indicated regions. Experiments were performed in triplicate. (**e**) Force magnitudes at different regions are decomposed into their parallel (//) and perpendicular components (⊥). The values shown are the average force measured from all pillars in the specified regions for a 1-h experiment. Experiments were performed in triplicate. (**f**) Example of force traces of a pair of pillars engaged in a force dipole at a negatively curved region. (**g**) Example of force traces of pillars engaged in the crawling mechanism at a positively curved region. Error bars indicate s.e.m. Samples are considered statistically different for *P*<0.05 in unpaired Student's *t*-test and are indicated by the star.

To further investigate the mechanical coupling between both mechanisms, we analysed cell–substrate interactions through focal adhesion (FA) distribution and traction forces. FAs appear as mechanosensitive units whose dynamics and orientation are regulated by traction forces^34^,^35^,^36^. In the case of wound healing, our recent findings showed that FAs were not only coupled to protrusive extensions towards the gap but also to actin contractile bundles^29^. We first analysed how the local curvature affected the distribution and the orientation of FAs. Large and mature FAs could be detected close to the edge of the gap, whereas a few microns away from the edge they were generally smaller and less dense (Fig. 3a, left). Furthermore, analysis of the FA orientation (Fig. 3a, right) revealed that the majority of them aligned tangentially to the gap edge in negative curvature regions (69.5% with their major axis oriented between 10° and 30° with respect to the edge). In positive regions, more FAs were perpendicularly oriented with respect to the edge (44.8% between 70° and 90°). This was confirmed using live-cell imaging of fluorescently labelled paxillin (Supplementary Fig. 5 and Supplementary Movie 4). We then investigated the relationship between traction forces and gap curvature. Flexible micropillar substrates^37^ were used to measure traction forces exerted by cells on the substrate during gap closure (Fig. 3b and Supplementary Movie 5). In agreement with the FA localization, the highest forces were concentrated at the gap edge (average force magnitude per pillar=19.98±0.82 nN, *n*=56 mean±s.e.m.), as compared with regions spaced >15 μm from the edge (10.25±1.01 nN, *n*=306, mean±s.e.m., *P*<0.05 in unpaired Student's *t*-test; Fig. 3d) as previously described^37^,^38^. Differences in force magnitude and orientation between positively and negatively curved regions could be noted (Fig. 3c,e). Analysis of the orientation of the forces showed that negative curvature promoted the formation of transient force dipoles parallel to the edge (mean=9.74±0.31 nN, *n*=1021, mean±s.e.m., maximum (max) value=56.01 nN; Fig. 3c,f). This force distribution can be attributed to purse-string mechanism with the cable coupled to the substrate through FAs and pulling inwardly from two sides as previously shown^29^. In contrast, the largest forces in positively curved regions were directed perpendicular to the edge (mean=17.78±0.4 nN, *n*=332, mean±s.e.m., max value=76.77 nN; Fig. 3c,g). To confirm the results obtained with flexible micropillars, we performed gap-closure experiments in MDCK cells using traction force microscopy on continuous deformable silicon substrates^38^,^39^,^40^. Similar force patterns were observed in such conditions including force dipoles at negatively curved regions and backward-pulling forces at positively curved ones (Supplementary Movie 6). Overall, these force distributions confirmed that a predominant crawling mechanism with perpendicular forces was observed in regions of positive curvature, whereas a purse-string mechanism with tangential forces with respect to the cell boundary was favored in negative regions (sustaining theoretical conjectures^31^,^41^,^42^).

### Large-scale mechanical coupling through actin cables

**Figure 4:**
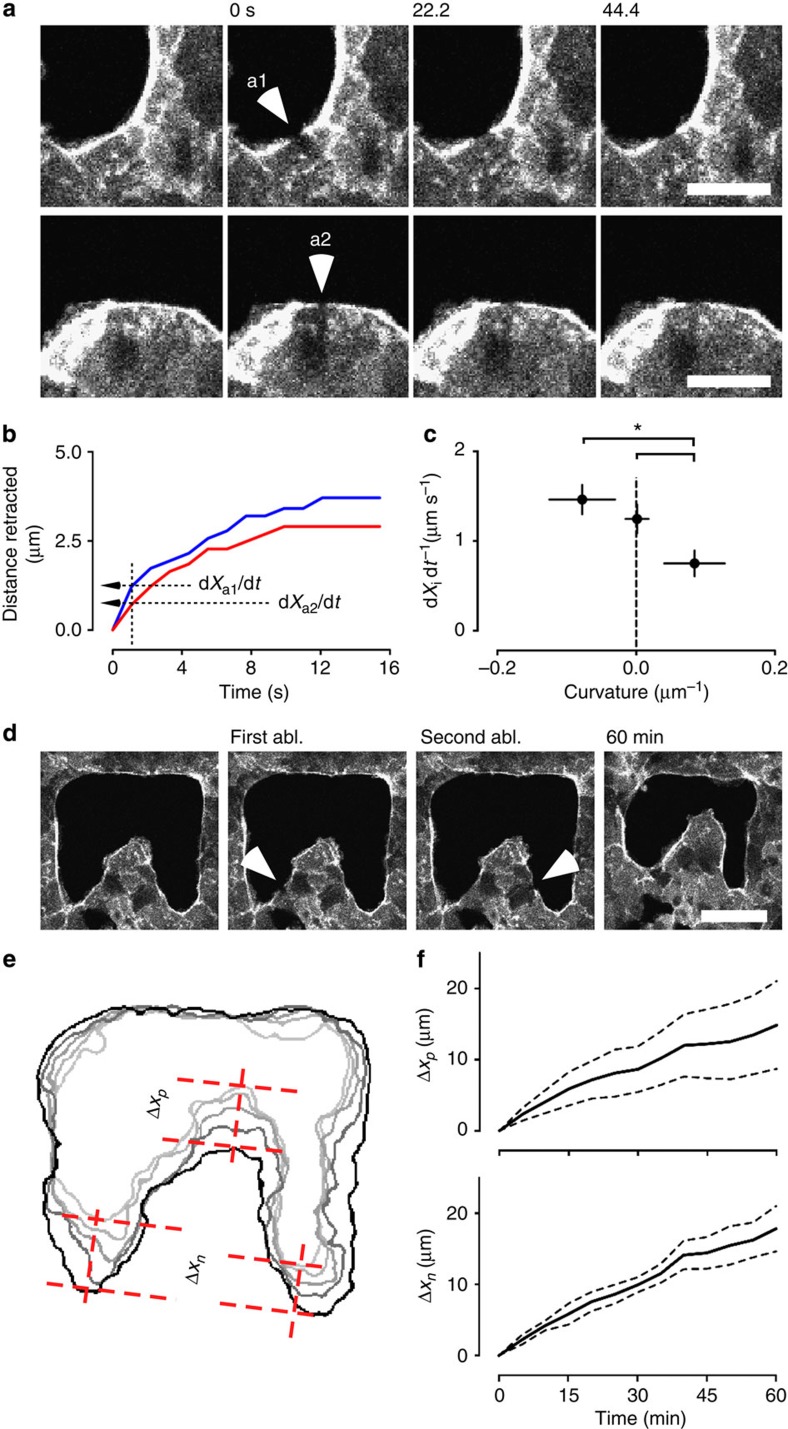
Laser ablation of the purse-string cable. (**a**) Examples of laser microsurgery at positive and negative curvatures. Retraction of the cable (relaxation of elastic tension) can be visualized over time. Arrowheads show the site of ablation. Scale bar, 20 μm. (**b**) Measurement of cable retraction after laser ablation as shown in **a**. Initial velocity of retraction (d*X*_*i*_ d*t*^−*1*^) is a function of the elastic tension. (**c**) Plot of the initial retraction velocity after ablation as a function of the curvature of the edge (*P*<0.05; *n*=76 independent regions from nine independent gaps). (**d**) Laser ablation of two sides of a positively curved region causes release in tension and allows crawling to advance the protrusion. Arrowheads show the site of ablation. Scale bar, 50 μm. (**e**) Overlays of the gap edge at different times after laser abaltion. *Δx*_p_ is distance crawled by the tip of the finger. *Δx*_n_ is the average advancement of the two negatively curved regions. (**f**) Top, advancement of the tip of the finger (*Δx*_p_) over time. Bottom, average distance travelled by the negative regions (*Δx*_n_) over time. Experiments were performed in quadruplicate. Error bars and dashed lines indicate s.e.m. Samples are considered statistically different for *P*<0.05 in unpaired Student's *t*-test and are indicated by the star.

Immunostaining of the actomyosin cable demonstrated continuity in actin organization along the edge of the monolayer from regions of negative to more positive curvatures, suggesting the existence of a mechanical coupling over multiple cells along the gap (Fig. 2a). To probe how tension forces were distributed at the edge, we used laser ablation to locally disrupt the actin cable at different regions (Fig. 4a). As a first approximation, tension of the cable could be assessed by measuring the recoil speed of the cable in response to the ablation (Fig. 4b). Analysis of the recoil speed (Fig. 4c) revealed that tension within the cable decreased as curvature increased from negative to positive, consistent with the observation of a thinner cable in positive regions, both *in vitro* and *in vivo* (Fig. 2 and Supplementary Fig. 4).

In addition, we employed laser microsurgery to test the mechanical connection between neighbouring regions of opposite curvature (Fig. 4d). Release of tension within the cable at the side of positive regions allowed the crawling mechanism to effectively move forward the whole finger-like structure (Fig. 4d–f, top) in a manner similar to leader cell-driven migration^22^,^37^. At the same time, displacement of negatively curved regions did not change as compared with control experiments (Fig. 4f, bottom). Our experiments show that crawling forces always point outwards from the tissue (that is, into the gap), thus helping closure at all curvatures, but particularly in positively curved regions. On the contrary, forces due to cable constriction drive the advancement of the edge in negatively curved regions, where they point outside of the tissue, but hinder forward movement in positively curved regions since they point backwards (away from the gap). Thus, our findings demonstrate that both mechanisms add up in negatively curved regions, making them advance faster than positively curved ones that are subjected to a tug-of-war between the two mechanisms.

### Effect of fibronectin and inhibition of motility mechanisms

**Figure 5:**
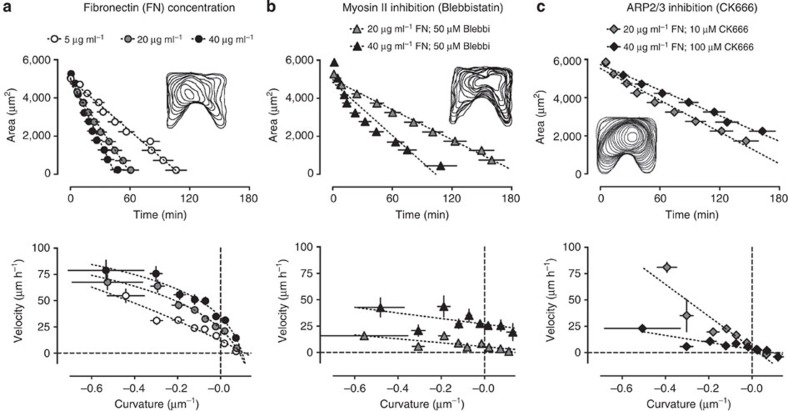
Fibronectin concentration and pharmacological inhibition influence cell-crawling and purse-string contraction. Top row, area decay of the gap over time. The linear fitting of experimental data is shown by the dashed line. Insets, overlay impression of outlines at different time points from representative experiments. Bottom row, relation between velocity–curvature. Logarithmic (best fit for **a**; *R*^2^=0.94-0.97) and linear fitting (best fit for **b**,**c**) of experimental data are shown by dashed line as guide of the eyes. (**a**) Closure of gaps on substrates coated with 5, 20 and 40 μg ml^−1^ fibronectin (*n*=15–19). Inset, representative experiment with 20 μg ml^−1^ fibronectin. (**b**) Blebbistatin inhibition of myosin II (50 μM) tested at 20 and 40 μg ml^−1^ of fibronectin (*n*=9–19). Inset, myosin II inhibition at 40 μg ml^−1^ fibronectin. (**c**) CK666 inhibition of the ARP2/3 complex (10 and 100 μM) tested at 20 and 40 μg ml^−1^ fibronectin (*n*=5–11). Inset, ARP2/3 inhibition at 10 μM CK666 and 20 μg ml^−1^ fibronectin. Error bars are s.e.m.

To further explore the mechanical coupling between crawling and purse-string mechanisms, we experimentally changed the strength of crawling and purse-string in our test system. First, we altered the cell-crawling mechanism by varying the amount of fibronectin absorbed on the substrate (Supplementary Fig. 2). At higher amounts of fibronectin (40 μg ml^−1^), a large number of lamellipodia drove a fast closure of the gap characterized by rough edges (Fig. 5a, top and Supplementary Movie 8). Importantly, the tip of the positive edge moved forward under the effect of enhanced crawling. Conversely, lower amounts of fibronectin (5 μg ml^−1^) slowed down the process. Smooth edges and the rounding of the gap indicated reduced crawling with the purse-string mechanism driving the closure. Finally, an intermediate situation could be noted at our standard conditions (20 μg ml^−1^), suggesting both mechanisms acting together in a balanced manner. In general, increasing strength of crawling was reflected by reduced curvature dependence. Furthermore, higher crawling induced a near constant velocity increase at all curvatures (Fig. 5a, bottom). Taken together, these results indicate that the crawling mechanism is weakly dependent on curvature. We then used pharmacological treatments to perturb either cell crawling or actin purse-string contractility. The inhibition of myosin II by blebbistatin decreased the tension within the cable and thus the cell-crawling mechanism becomes dominant for gap closure (Fig. 5b). Under these conditions, we observed that the slope of the velocity–curvature relationship for negative curvatures is greatly reduced as compared with control conditions (Fig. 5a). Furthermore, an increase in fibronectin concentration under myosin II inhibition induced a similar trend of the curvature–velocity relationship with higher velocity values at all curvatures. As consequence of a reduced mechanical coupling between negative and positive regions through the actomyosin cable, inhibition of myosin induced the tip of the positive region to crawl forward (Fig. 5b, inset) similar to that in our laser ablation experiments (Fig. 4d–f). Overall, we observed that reduced purse-string contractility released the crawling motion of the positive regions. Finally, we examined the role of the purse-string on epithelial gap closure. To do so, we inhibited cell crawling by hampering actin polymerization with the Arp2/3 inhibitor CK666. As expected, such inhibition prevented cell-crawling leading to a smoothing and rounding of the gap shape (Fig. 5c, inset). Under these conditions, we observed that in negative regions the front edge velocity depended more on the curvature (Fig. 5c). Interestingly, the velocity of highly negative regions was less affected by Arp2/3 inhibition, indicating once again that the purse-string was the most important mechanism in these regions. Conversely, we did not observe any displacement of the tissue towards the gap at zero curvature (flat regions) and a retraction at positive curvatures leading to negative velocities. These motions should be a direct consequence of hindering lamellipodia-driven crawling, which is dominant for positive curvature regions. Altogether, these experiments indicate that the edge speed due to purse-string depends on the curvature of the tissue edge, whereas the one due to cell crawling is nearly constant at different curvatures.

### Mathematical model

To have a deeper understanding of the underlying mechanisms, we constructed and simulated a mathematical model based on the experimental results. The homophilic interaction of cells within the tissue was modelled as a viscous interaction that exerts a drag on the movement of the gap. The constitutive equation of the monolayer can be written as:


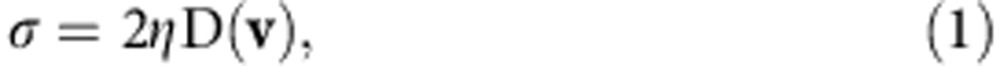


where *η* is the viscosity; *D*(*v*)=1/2(∇**v**+∇**v**^T^) is the symmetrized part of the velocity gradient. The interaction of the cells with the substrate is considered as a frictional resistance to the movement proportional to the velocity. The momentum balance expresses the balance between cell–cell and cell–substrate interactions in the bulk of the tissue:


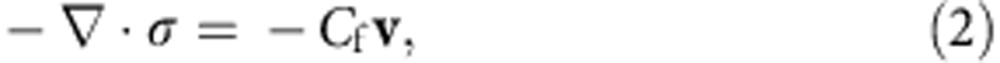


**Figure 6:**
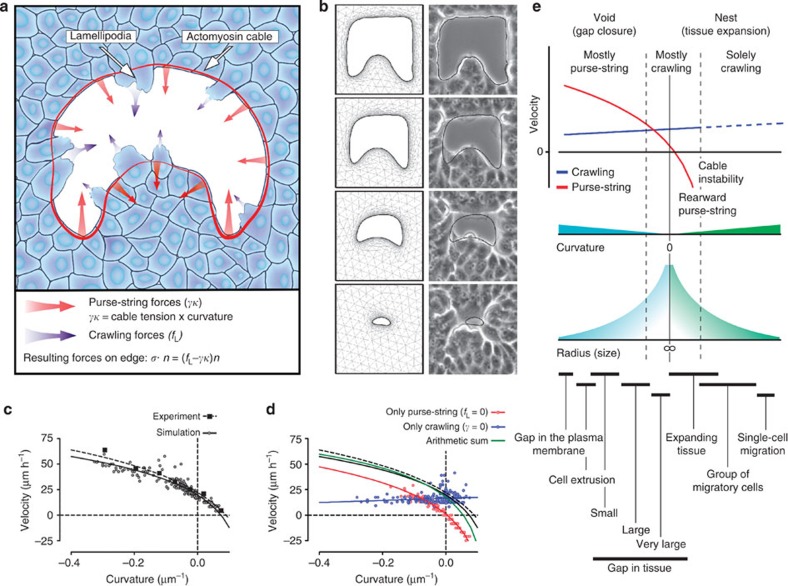
Simulation and mathematical model. (**a**) Cartoon showing the model of the mechanical coupling between purse-string and crawling mechanisms. Red and blue arrows indicate the direction and magnitude of the local stress induced by purse-string and crawling, respectively. Stress pulling the edge of the gap results from the arithmetic sum of purse-string and crawling stresses. (**b**) *In silico* simulation of gap closure. Cell–cell interactions throughout the tissue are modelled as viscous drag. Cell–substrate interaction contributes as a frictional component. Stresses at the edge are as in **a**. (**c**) Comparison of experimental and simulation results. Lines indicate logarithmic fits (best fit; *R*^2^=0.88–0.97) of the data points as a visual guide. (**d**) Simulation showing the contribution of crawling and purse-string mechanisms separately. Red (only purse-string) and blue (only crawling) lines are logarithmic and linear fits, respectively. The green line is the arithmetic sum of red and blue fits. Line fittings from **c** (black lines) are displayed for comparison. (**e**) Line fittings from **d** are used to illustrate the relationship between local curvature, gap size and the closure mechanism.

Where *C*_f_ is a friction coefficient. Finally, inspired by the theoretical assumptions of our previous models^26^,^42^ and using information obtained from our experimental results, we considered the force pulling on the gap boundary as the sum of two independent components: the crawling and the purse-string (Fig. 6a)^24^,^25^. Thus, the normal forces acting on the moving boundary (∂*W*) of the tissue are modelled as a boundary condition for the stress tensor:


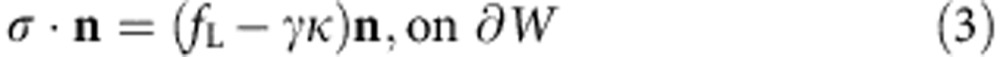


Where *n* is the local normal vector on the boundary, directed towards the exterior of the tissue, *f*_L_ is the density of forces exerted by the lamellipodia (per unit length), *κ* is the local curvature whose sign is defined so that circular gaps have negative curvature and finally *γ* is the tension exerted by the actomyosin cable (purse-string). The negative sign in front of the tension term means that in positively curved regions the purse-string pulls the edge backwards (towards the interior of the tissue) and thus works against cell crawling. This makes these regions move slower than negatively curved ones where the two mechanisms add up to make the edge advance into the gap.

For simplicity and in agreement with our previous theoretical models^26^, we suppose that neither the cable tension nor the cell-crawling force depend on curvature, leaving a more detailed and complex description for the future. This enables us to have few parameters to fit and to separate in a clear way the contribution of the two mechanisms. Numerical solution of the system of equations (1) and (2) coupled with the boundary conditions (3) on the gap edge was performed by finite element analysis. The initial contour of the edge was extracted from segmentation of particular experiments. Numerical simulations performed as described in the Supplementary material were sufficiently accurate in recapitulating the evolution of the edge (Fig. 6b and Supplementary Movie 10) and the velocity–curvature relation reflected the one that was observed in *in vitro* experiments (Fig. 6c). Figure 1e shows a good linear fit with slope directly proportional to the cable tension^24^, which is valid close to zero curvature. However, speed is lower than that predicted by this linear fit at more negative curvatures, where the cells suffer the nonlinear effect of the tissue viscous drag. The resulting velocity–curvature relation could be empirically represented by a logarithmic decay (Fig. 6c). Solution of the simulation for zero crawling or zero purse-string shows the behaviour of advancement of the edge if one or the other mechanism acts alone (Fig. 6d and Supplementary Movie 11).

Apart from the remarkable agreement between simulations and experimental observations (Fig. 6c), our modelling approach allows us not only to clearly separate the relative contributions of the two mechanisms but also to determine how they act in synergy to yield the overall movement of the edge (Fig. 6d).

## Discussion

Epithelial tissues are subjected to large-scale reorganization during morphogenesis, wound closure or epithelial–mesenchymal transition^43^. Such remodelling involves ‘epithelial plasticity' in which cell–cell interactions play a major role and border cells may become motile^44^,^45^. In this context, the closure of epithelia is of particular interest since it recapitulates these different scenarios. Classical views of epithelial gap closure involve either lamellipodium crawling or actomyosin-based purse-string contraction depending on the biochemical and physical properties of the environment^7^. In agreement with previous observations^10^,^19^,^27^, our study shows that these two mechanisms do not exclude each other but, on the contrary, largely coexist. Furthermore, our investigation demonstrates that the two mechanisms can mechanically influence each other and are tightly regulated through the geometry of the tissue. As such, cell movement at the gap edge relies on a universal coupling between local curvature and actin organization, which drives a large-scale process linking cell crawling and actin-based purse-string mechanisms. Positively curved regions show high lamellipodia activity and exerted forces on the substrate perpendicular to the edge that may promote a net forward movement of the cells. However, these forces are counteracted by rearward forces because of the contractility of actomyosin bundles, thus hindering cell advancement in the presence of backward-pulling purse-string cables. Importantly, once the tension within this actomyosin cable is released by either inhibition of myosin II or laser ablation, cell crawling is enhanced independently of the curvature. Negative regions are characterized by thick actomyosin cables that exert tangential force dipoles (purse-string mechanism). Within these regions, purse-string contraction positively pairs with cell-crawling, resulting in high velocities. These curvature-specific closure phenotypes and the general organization of actin structures are reproduced *in vivo* for wounds in the notum epithelia of Drosophila pupae, indicating that the mechanisms driving gap closure are universal. Furthermore, our experimental results proved that crawling is present at all curvatures but is most effective at flat and positive curvatures. Conversely, purse-string dominated the advancement of highly negative curvatures, but was ineffective at zero curvature and counterproductive at positive ones because of its rearward direction.

These experimental results are well recapitulated by *in silico* simulation. Our model's decomposition of the two mechanisms shows that the speed of edge advancement due to crawling is nearly constant at all curvatures with a slight tendency to increase with curvature. On the other hand, the velocity due to purse-string contraction decreases with increasing curvature (that is, high velocity at more negative curvatures). As a result of the mechanical coupling between the two mechanisms, the overall advancement of the edge also decreases with curvature.

The relative value of the contributing mechanisms reflects the observation that purse-string and crawling are contributing to different extents at differently curved edges (Fig. 6e). Interestingly, actin structures driving the two mechanisms (that is, branched actin and actin fibres, respectively) have been observed to self-assemble in response to the curvature of the cell membrane in cytoplasm fragments^46^, suggesting that curvature of the cell–tissue boundary and type of motility mechanism are fundamentally correlated. Furthermore, such actin structures at the base of lamellipodia crawling and purse-string constriction seem to be antagonistic through a mechanism controlled by Rho and Rac^17^,^47^. However, in our experiments, we observe the coexistence of such structures, suggesting for a regulatory mechanism, which unfortunately remains elusive at this point. To better understand the interaction and regulation occurring between the two mechanisms, we use our mathematical model. Indeed, the decomposed contribution of the two mechanisms suggests that cells have evolved to select the mechanism that drives the closure in an efficient way^31^,^41^,^42^. This representation explains the prevalence of one or the other closure mechanism depending on the local curvature and, more generally, on the size of the gap. As small gaps (for example, cell extrusion^3^,^4^,^20^) are highly curved, they may predominantly close by purse-string constriction. Similarly, gaps of subcellular scale on the plasma membrane typically close via constriction of an actomyosin ring^6^,^48^. This mechanism loses importance as the radius of the gap increases and, if enough ECM is present, large gaps close by crawling on the substrate^9^. Generally, *in vitro* assays to study wound closure—scratch and wound model assays—generate very large gaps with nearly flat edges that indeed have been reported to advance by crawling^16^,^38^. This can be explained by the purse-string being ineffective in flat regions since it does not produce normal forces to help cells advance into the gap. Interestingly, lamellipodial crawling at local regions of positive curvature helps to create an unstable front, and finger-like protrusions form in this scenario^19^,^49^,^50^. Cells at the tip of these protrusions show enhanced crawling capacity and are thought to drag neighbouring cells using actin bundles analogous to the two continuous actomyosin cables we observed (Fig. 2d) extending behind tip cells through the flat edge of the gap^17^. In fingers and tip structures, this should generate a tug-of-war between the crawling and the purse-string mechanisms, with purse-string forces resisting the advancement of the ‘leader' cell. Indeed, this was observed in migratory tissue in model wound experiments (Supplementary Fig. 6 and Supplementary Movie 12). When crawling forces are sufficiently large, this tension may result in pulling forces dragging the connected neighbouring ‘follower' cells. Similarly, positive curvatures, which characterize expanding tissues, are likely to perceive a force balance between the two mechanisms. However, we have observed that the cable is apparently weaker and might become unstable at positive curvatures, and at high positive curvatures this cable may break down. Thus, in the long term this instability and the proliferation of cells within the tissue may direct this equilibrium in favour of the crawling mechanism^28^,^32^. Importantly, this type of equilibrium can be seen in colonies of cancer cells. Once the equilibrium is broken, for example, by reduction of cell–cell junctions and the consequent decrease in tension within the cable, cells at the boundary are free to ‘crawl away' and metastatically invade the surrounding tissues^18^,^51^. At even higher curvatures that distinguish small groups of cells advancing under the effect of a chemoattractant or even single cells, the purse string is not present leaving crawling as the sole mechanism of motility^52^. Overall, our data and analysis provide a general interpretation of the relation between epithelial tissue motility and its geometrical cues.

## Methods

### Cell culture and reagents

MDCK cells were cultured in DMEM (Life Technologies) supplemented with 10% fetal bovine serum (FBS; Life Technologies). MDCK cells stably expressing GFP-Actin were kindly provided by J.W. Nelson. Stably transfected cells were cultured in DMEM with 10% FBS and Geneticin (100 μg ml^−1^; Life Technologies). Cells were cultured at 37° C in a humidified incubator with 5% CO_2_ and subcultured every 2/3 days using the Trypsin/EDTA (Life Technologies) method. Blebbistatin (Sigma-Aldrich) and CK666 (Sigma-Aldrich) were dissolved in dimethyl sulfoxide at a concentration of 1 mM before being added to the media. Fibronectin (Sigma) was reconstituted in deionized water at a concentration of 1 mg ml^−1^. Dilution to working solution was also performed in deionized water. Transient transfection with mApple-paxillin (generated in the laboratory of M.W. Davidson) on stable GFP-Actin MDCK cells was performed using electroporation (Neon Transfection system; Life Technologies).

### Preparation of PDMS stencils

Stencils made of PDMS (Dow-Corning) to create gaps in the epithelium have been produced by a two-step molding process as previously described^9^,^40^ with minor variations. Briefly, shapes of desired dimensions and geometries were transferred from a mask on a silicon wafer by photolithography. Deep reactive ion etching technique was used to create pillars of desired height (∼100 μm) emerging from the wafer. After silanization of the wafer by vapour deposition (1H, 1H, 2H, 2H-Perfluorooctyl-trichlorosilane; Sigma), PDMS at a ratio 1/10 (curing agent/base) was crosslinked on the wafer (80° C for 2 h) to produce a mold having holes on its surface. After removal from the wafer, this second mold was silanized and the PDMS crosslinking (80° C for 2 h) was repeated to produce stencils in place of the holes.

### Preparation of wound-free gaps in the MDCK-confluent epithelium

In all, 600 μl of varying concentration of fibronectin solution was incubated for 1 h at 37° C on a glass-bottom Petri dish (IBIDI, Sciencewerke Pte Ltd). Thereafter, excess of protein still in solution or weakly bound was washed away three times with deionized water. The solution was removed and the substrate was allowed to dry. A PDMS cut with stencils emerging from its surface was carefully placed upside-down on the glass-bottom Petri dish and incubated for 1 h at 37° C and 100% rH. Thereafter, 7 μl of 0.2% pluronic acid solution (Sigma) were flown in between the PDMS stencils by capillarity to passivate the sides of the stencils. After 1 h incubation at 37° C, pluronic acid was washed away three times with deionized water. The device was again dried to allow cell suspension (7 μl with ∼100,000 cells) to flow by capillarity underneath the PDMS block in between the stencils. To prevent washout, cells were allowed to adhere to the substrate for 30–45 min in a cell culture incubator. DMEM media (400 μl) supplemented with 10% FBS were added to supplement cell with sufficient nutrients. Cells were grown for 12–18 h under appropriate culture conditions to permit formation of a mature epithelium around the stencils. Homogenous distribution of protein on the substrate at the onset of the experiment was verified by mixing fluorescently labelled fibronectin with unlabelled one (ratio 1/10 w/w; Supplementary Fig. 3). Previous report demonstrated that fibronectin concentration remains unaltered during the experimental time^9^.

### Live-cell imaging

Samples prepared as described in the previous section were placed on microscope stage. After careful removal of the stencils and replenishment of media with fresh DMEM pre-equilibrated for temperature and CO_2_, dynamics of gap's closure were visualized with phase contrast microscopy using BioStation IM-Q imaging system (Nikon) equipped with × 20 phase objective. Dissolved inorganic carbon (DIC) and brightfield fluorescence as well as force measurements have been performed using a fully automated IX81 inverted microscope (Olympus) equipped with oil-immersion objectives ( × 20 and × 40), appropriate filter sets, lamp and camera. All live-cell experiments have been conducted at 37̊ C and 5% CO_2_. For inhibition experiments, samples were pre-incubated with the drugs at the reported concentration for 1 h. Drug concentrations were kept constant also during washing and the entire experimental time.

### Image segmentation and analysis of gap-closure experiments

The image segmentation of phase contrast and DIC images was performed using the in-house MATLAB software. The algorithm applies a range filter on the raw image. The range filter gives high response at region with large intensity variations and low response at region with homogeneous intensity. In our image, the homogeneous region corresponds to the epithelial gap, while large variation arose from cellular components. The size of the window used varied from 7 × 7 to 13 × 13 pixels. The range-filtered image was then thresholded and refined by morphological operations to generate a binary mask corresponding to the gap. After segmentation, the contour of the epithelial gap was extracted from the binary mask. Randomly selected points along the contour were analysed for curvature and velocity. Between 6 and 8 points per gap were selected to cover the whole spread of curvatures within the gap. For each point, local curvature was computed with respect to neighbouring points spaced 15 μm away on both sides.

### Immunofluorescence

After careful removal of the stencils, samples were washed with media equilibrated for temperature and CO_2_ and placed back in the incubator for specified times to allow cell migration. Thereafter, samples were washed with PBS with calcium (Life Technologies) and fixed for 15 min with paraformaldehyde (4% in PBS with calcium). Paraformaldehyde was inactivated by incubating the samples with NH_4_Cl (10 mM in PBS) for 15 min. Following three washes with PBS, samples were incubated with 1% BSA (Sigma) and Triton X-100 (0.1%; Sigma) for other 15 min. For staining of phosphomyosin light chain, samples were incubated with primary antibody against phosphomyosin light chain 2 (Ser19; Cell Signalling Technology; cat. no. 3675) raised in rabbit (1:50 dilution) and cortactin (P80/85; Millipore; cat. no. 05–180) raised in mouse (1:100), washed with PBS and incubated for 1 h with Alexa fluor 488 donkey anti-rabbit secondary antibody (1:100 dilution; Life Technologies) and Alexa fluor 568 donkey anti-mouse (1:100 dilution; Life Technologies). For staining of paxillin, samples were incubated with primary antibody against paxillin raised in mouse (1:100 dilution; Abcam; cat. no. ab3127), washed with PBS and incubated for 1 h with Alexa 488 goat anti-mouse secondary antibody (1:100 dilution, Life Technologies). For both immunofluorescence, samples were then washed and stained with 4,6-diamidino-2-phenylindole (1 μM; Sigma), and for actin they were stained with phalloidin Alexa fluor 647 (1:100; Life Technologies) or TRITC-phalloidin (1:50; Sigma). Images were acquired using confocal microscopy (LSM710, Zeiss or Nikon A1R, Nikon) with oil-immersion × 100 objective. For high-resolution images of focal adhesion orientation (Paxillin)-structured illumination microscopy in the total internal reflection fluorescence (TIRF) mode was employed (N-SIM, Nikon) using an oil-immersion × 100 objective.

### Force measurements

Traction forces exerted on the substrate have been accessed by two independent methods: deflection of pillars made of elastomeric silicon (elastic micropillar experiments)^37^,^53^ and by deformation of a soft elastic substrate (traction force microscopy)^29^,^38^,^39^,^40^. Pillars made of PDMS have been produced by soft lithography and their top functionalized with fibronectin^54^. PDMS cut with stencils on top was carefully placed upside-down on top of the pillars. Passivation of sides of pillars and stencils was achieved by addition of 10 μl of pluronic acid (0.2%) at the side of the PDMS' cuts. Pluronic solution was allowed to enter by capillarity in the space between the pillars and the stencils. After multiple washing with water and once with medium, cell suspension was added on the sides of the PDMS. With gentle pipetting, cells could diffuse in the space between the two PDMS cuts. Traction force microscopy was performed by detecting displacement of beads embedded on the surface of a soft silicon elastomer. Briefly, CyA and CyB (Dow-Corning) components were mixed in a 1:1 ratio, spin-coated on a glass-bottom dish at 500 r.p.m. for 1 min and cured at 80 °C for 2 h (elastic modulus ∼8 kPa). The substrate was silanized using a 5% solution of (3-Aminopropyl) trimethoxysilane (Sigma) in ethyl alcohol for 5 min. Subsequently, carboxylated green fluorescent beads (100 nm, Invitrogen) were diluted in deionized water (1:500) and added to the substrate. After incubation for 5 min, the substrates were washed with deionized water to remove loosely bound beads. Substrates were coated with fibronectin at a concentration of 20 μg ml^−1^ for 30 min at 37° C. Stencils were incubated in pluronic solution (0.2%) to avoid adherence to the soft silicon surface. After washing with deionized water, stencils were carefully placed on the soft surface. Thereafter, cells were injected by capillarity in between the stencils. Cells were grown for 12–18 h under appropriate conditions to permit formation of a mature epithelium around the stencils. After removal of the stencils, live-cell imaging of closure of the gap and simultaneous detection of deflection of pillars (elastic micropillar experiments) or displacement of beads (traction force microscopy) using a fully automated Olympus IX81 inverted microscope equipped with oil-immersion × 20 and × 40 objectives. Analysis of forces by pillar deflection or bead displacement was performed as previously described^29^,^53^.

### Laser microsurgery of actomyosin cable in MDCK cells

Laser microsurgery of the actomyosin cable at the gaps' edge was performed using an ultraviolet laser (355 nm; 300 ps; PowerChip, Teem Photonics) mounted on a Nikon A1R confocal microscope with a × 60 oil-immersion objective. Ablation of the actomyosin cable was performed by moving the laser beam at low power (10 nW at the back aperture of the objective) with low vectorial speed perpendicularly to the edge. Integrity of the cell after ablation was visually verified with brightfield illumination. Images were acquired every 1.1 s and analysis of speed of recoil^55^ and movement of the gaps' edge was performed in ImageJ.

### Laser ablation of *Drosophila melanogaster's* notum epithelium

The *in vivo* experiments were performed in *D. melanogaster*. Flies were maintained on standard conditions and kept at 25° C. The UAS/GAL4 system^56^ was used to follow the dynamics of wound closure. Flies with w;; pnr-GAL4 (ref. 57) were recombined with w;; UAS-mCherry-Moesin^58^ to obtain w;; pnr-GAL4, UAS-mCherry-Moesin/TM6b. Flies with w;spaghetti-sqh-GFP were used to follow myosin dynamics^59^,^60^. Pupae with these genotypes were staged at 13 h after puparium formation and their notum epithelium was imaged at 25° C on an Andor Revolution XD spinning disk imaging system (Nikon Eclipse Ti-E and Yokogawa CSU-x1) with a × 60 oil-immersion objective. Pupa-mounting procedure and visualization were performed as previously described^59^. Complex laser wound shapes were achieved using a MicroPoint (Andor Technology) ultraviolet nitrogen-pumped dye laser (435 nm). To ablate the desired shape, *xy* coordinates (11 coordinates) were manually selected on the iQ 2.5 controller software (Andor Technology). Laser was triggered manually on each coordinate with 15 pulses at maximum power. Imaging started immediately after the wounding protocol. Actin cable intensity was obtained by manually selecting regions of interest of ∼2.5 mm at the gap edge.

### *In silico* model

The continuum mechanics framework provides the set of partial differential equations to describe the system with respect to time and space as follows:

The tissue is modelled as a viscous material: its stress tensor *σ* is given by =2*η***D**(**v**)−*pI*, where *η* is the viscosity; **D**(**v**)=1/2(∇**v+**∇**v**^**T**^) is the symmetrized part of the velocity gradient, *p* is a pressure and *I* is the identity matrix. Cells are considered as a tridimensional incompressible material; however, in the bidimensional configuration studied here, where the height of cells is negligible compared with the size of the monolayer, cells can adjust their height by spreading or contracting: as a two-dimensional (2D) material, the tissue is compressible and any vertical deformation is compensated by the pressure term. The 2D constitutive equation of the monolayer can be written with effective viscosity (still denoted as *η* for the sake of simplicity): **σ**=2*η***D**(**v**).

Cell–substrate interaction forces are modelled as an external friction force proportional to velocity; the momentum balance expresses the balance between cell–cell and cell–substrate interactions. It leads to the following formula: −∇*.σ*=−*C*_f_**v**, where *C*_f_ is a friction coefficient.

The closing mechanisms acting on the border ∂*W* of the tissue are modelled as a boundary condition for the stress tensor: *σ*.**n=−**(*f*_L_−*γκ*)**n**, on ∂*W*, where *n* is the normal vector on the boundary, directed towards the exterior of the tissue, *κ* is the local curvature, by convention negative in case of a circular gap for instance, *γ* is the tension exerted by the actomyosin structure and finally, *f*_L_ is the lineic density of forces exerted by the lamellipodia.

In the present set-up, the difficulty is to take into account the moving edge of the tissue and the fact that the motion is driven by forces exerted on it. Moreover, the expression of the forces depends on the geometrical properties of the edge. Such a situation is reminiscent of multiphase fluid modelling, for example, gas bubbles in a liquid, where the interface between two phases evolves in time according to their mechanical properties and the surface tension characteristic of this interface. The resulting partial differential equations can be numerically solved with appropriate methods such as the Level-set methods^61^, where the moving boundary is implicitly represented as the zero level-set of a function. The motion of the interface is described by the transport of the level-set function by the velocity field of the phases^26^; geometrical properties such as curvature and normal vectors can be extracted from the expression of the level-set function. For this study, we have set up a numerical framework inspired by works such as ref. 62, in which the computational domain is fixed, and the forces on the boundary appear as a source term in the momentum balance equation.

In our implementation, the time is discretized with a time step, Δ*t*, and the space is discretized with a triangular mesh that is adapted to the contour of the tissue at each time step; this mesh adaptation is essential to compute precisely the forces on the edge. The numerical algorithm is implemented as follows: at the initial step *t*=0, the initial contour of the tissue is extracted by hand from an image using the software ImageJ, with ∼50 points. A first level-set function is computed from it and an initial mesh is constructed with an adaptation criterion based on the level-set function^63^ with the use of the BAMG software ( https://www.ljll.math.upmc.fr/lehyaric/freesoft/bamg.htm). Then, in the following steps, and as long as the area of the gap is not zero, we compute the local curvature from the level-set function, the forces on the moving edge and the velocity field resulting from the momentum balance equation (solving this equation using a finite element method with the C++ library RHEOLEF^64^). Using the matlab toolbox ToolboxLS^65^, we transport the level-set function on the time interval [*t*,*t+*Δ*t*] along this velocity field and renormalize it. We then construct a mesh adapted to the new level-set function with the same method as in the initialization and, finally, the time step (*t*←*t*+Δ*t*) is incremented.

### Image analysis and statistics

Images were analysed with the in-house MATLAB software and ImageJ. Data analysis, statistics, graph plotting and fitting were performed using the MS Excel (Microsoft) and Prism (GraphPad) software. Statistical analysis was performed by unpaired Student's *t*-test. The significance threshold was set for the *t*-test as *P*<0.05. For all data sets, experiments were repeated in independent triplicates or more. Graphs display mean±s.e.m. unless otherwise specified. Mathematical modelling and *in silico* simulation were performed in-house using MATLAB and C++ codes as described in the Supplementary Material.

## Additional information

**How to cite this article:** Ravasio, A. *et al.* Gap geometry dictates epithelial closure efficiency. *Nat. Commun.* 6:7683 doi: 10.1038/ncomms8683 (2015).

## Supplementary Material

Supplementary Figures Supplementary Figures 1-6

Supplementary Movie 1 Closure of in-vitro gaps of different shapes. Life cell imaging of gap closure experiments shown in Fig. 1 B. Tracking over time of randomly chosen point and the edge of the gap are used to illustrate the different speed at different curvatures. Frame rate = 1 frame every 5 minutes.

Supplementary Movie 2 Live cell imaging of GFP-actin transfected cells. DIC (left) and brightfield fluorescence (right) of an exemplary gap closure experiment shown in Fig. 2 C. Frame rate = 1 image every 30 sec.

Supplementary Movie 3 Closure of wounds with varying geometries. Exemplary illustrating the laser microsurgery experiments of notum epithelium shown in Fig. 1 I. Frame rate = 1 image every 5 min.

Supplementary Movie 4 Live cell imaging of GFP-actin and m-cherry paxilin transfected cells. Brightfield fluorescence of actin (green) and TIRF of m-cherry paxilin illustrate the evolution of foal adhesion during a gap closure. Frame rate = 1 image every 5 min.

Supplementary Movie 5 Exemplary gap closure experiment combined with micropillar force measurements. Live cell imaging and traction force measurement shown in Fig. 3 B. Left, merged image of GFP-actin (green) and Cy3 fibronectin (red) staining the top of the pillars shows the advancement of the epithelium and simultaneous displacement of the top of the pillars. Middle, Cy3 fibronectin is shown separately to fully appreciate the displacement of the pillars. Right, vectorial representation of forces demonstrates the evolution of their orientation and magnitude over time. Frame rate = 1 image every 5 min.

Supplementary Movie 6 Traction force microscopy during gap closure. Merge of DIC image with vectorial representation of the deformation of a continuous soft substrate (Traction Force Microscopy). Frame rate = 1 image every 5 min

Supplementary Movie 7 Laser ablation of the actomyosin cable releases the tension and allows crawling forward of positive regions. Live imaging of GFP-actin shows the laser ablation of the cable at the two sides of the positive regions. During ablation (first part of the movie), the frame rate is 1 image every 1.1 sec. During the second part of the movie, gap closure is followed for 1 h at the frame rate of 1 image every 5 min. Frame rates are indicated in the movie. Site of ablation are highlighted by a white circle.

Supplementary Movie 8 Exemplary gap closure at different amounts of fibronectin. Phase contrast of exemplary gap closure experiments at the indicated concentration of fibronectin. Frame rate = 1 image every 5 minutes.

Supplementary Movie 9 Myosin and Arp2/3 inhibition experiments. Phase contrast of exemplary gap closure experiments at the indicated inhibitory conditions. Frame rate = 1 image every 5 minutes.

Supplementary Movie 10 Comparison between in-vitro gap closure experiment and simulation based on mathematical model. The two movies correspond to Fig. 6 in the article. Parameters of the model were chosen so that both the closing time and the overall shape would match.

Supplementary Movie 11 Comparison between different analytical solutions of mathematical model. These movies illustrate two limits of the model: either crawling dominant (middle) where the edges are advancing faster than the corners which eventually leads to sharpen the shape of the gap; or conversely pursestring dominant (right) where the corners are advancing faster, resulting in a much smoother shape. An intermediate scenario is seen when both mechanisms are set with similar magnitudes (left).

Supplementary Movie 12 Migration of MDCK in a model wound experiment. Movie showing tug-of-war between crawling and purse-string mechanism in a wound model experiment (see Suppl. Fig. 6). Substrate was coated with 20 μg/ml fibronectin. Frame rate = 1 image every 30 min.
