# Supplementary material for: Gap geometry dictates epithelial closure efficiency
Source: Nat Commun. 2015 Jul 9;6:7683. doi: 10.1038/ncomms8683 (PMC4510701; doi:10.1038/ncomms8683)
Supplement: Supplementary Figures — 1-6 [file ncomms8683-s1.pdf]

Supplementary figures

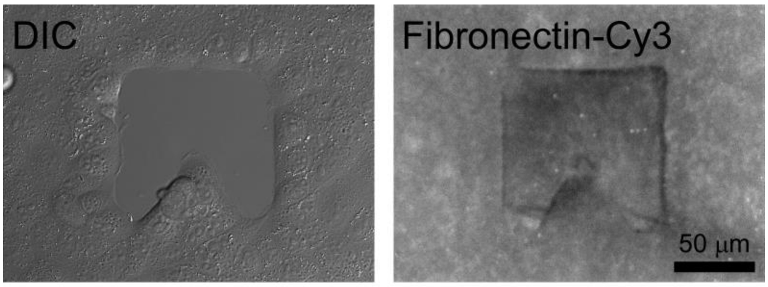

Supplementary Fig. 1 Ravasio et al.

Suppl. Fig. 1 – Fibronectin coating

Glass bottom petri dishes were coated with fibronectin mixed with covalently bound Cy3-fibronectin (10:1). After removal of the stencil, an overall homogenous distribution of the ECM can be observed within the gap area with only a thin line (< 1µm in average) of lower fibronectin being localized around the gap’s edge.

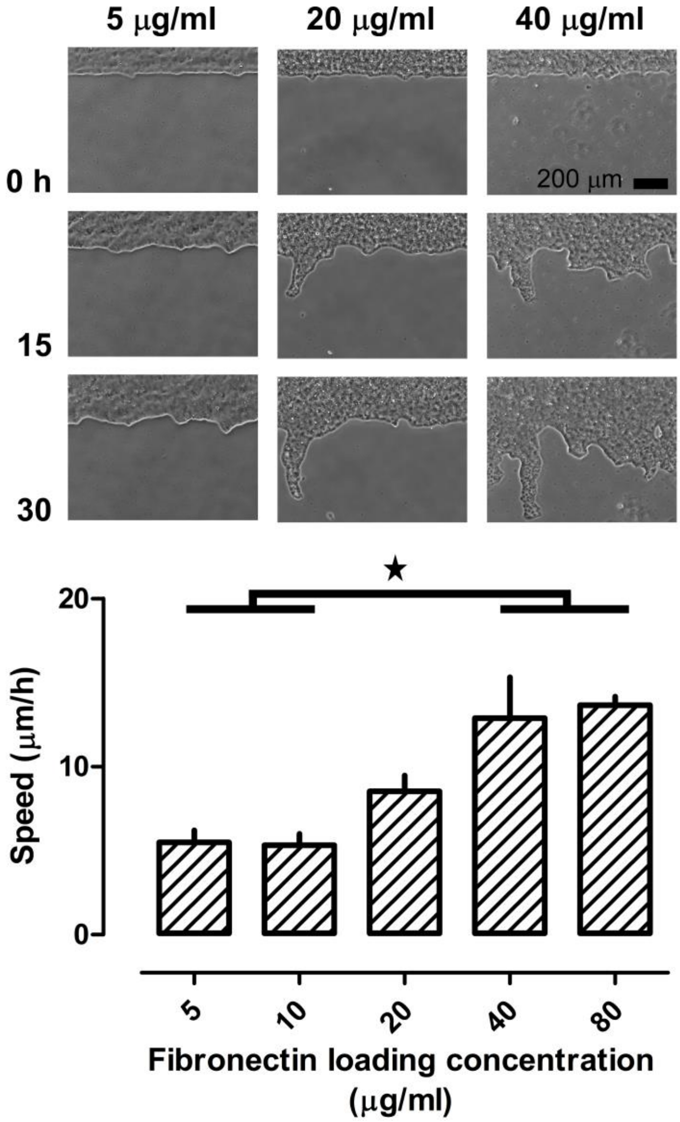

Supplementary Fig. 2 Ravasio et al.

Suppl. Fig. 2 – Effect of different fibronectin concentrations on speed of crawling

Top, time lapses of exemplary model wound experiments on glass bottom petri dishes coated with different amounts of fibronectin. Bottom, quantification of the speed of advancement of the tissue as a function of the amount of fibronectin. Dose response in migration speed was correlated to a larger number of lamellipodia, indicating that higher amounts of surface fibronectin increased crawling mediated migration.

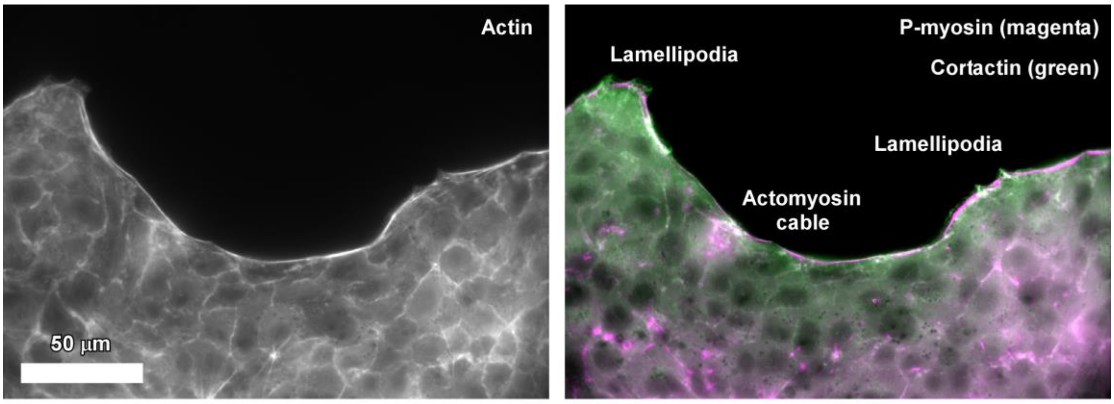

Supplementary Fig. 3 Ravasio et al.

Suppl. Fig. 3 – Immunofluorescence of tissue free edge in model-wound

Left, phalloidin staining of actin. Right, merged immunostaining for phosphorylated myosin light chain (magenta) and cortactin (green). An actomyosin cable was detected at negatively curved regions, whereas cable instabilities at positive curvatures promoted the formation of lamellipodia.

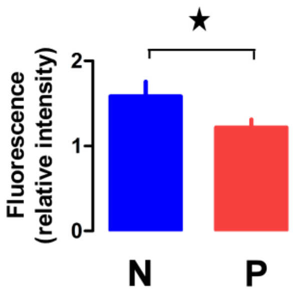

Supplementary Fig. 4 Ravasio et al.

Suppl. Fig. 4 – Analysis of actomyosin cable in wounds made in Drosophila notum epithelium

Actin cable (UAS-cherry-moesin flies) were used to assess cable intensity at regions of negative (N) and positive (P) curvatures. Fluorescence signal is higher at negative curvature, indicating for a thicker actin cable within this region. (p=0.0286; n = 4).

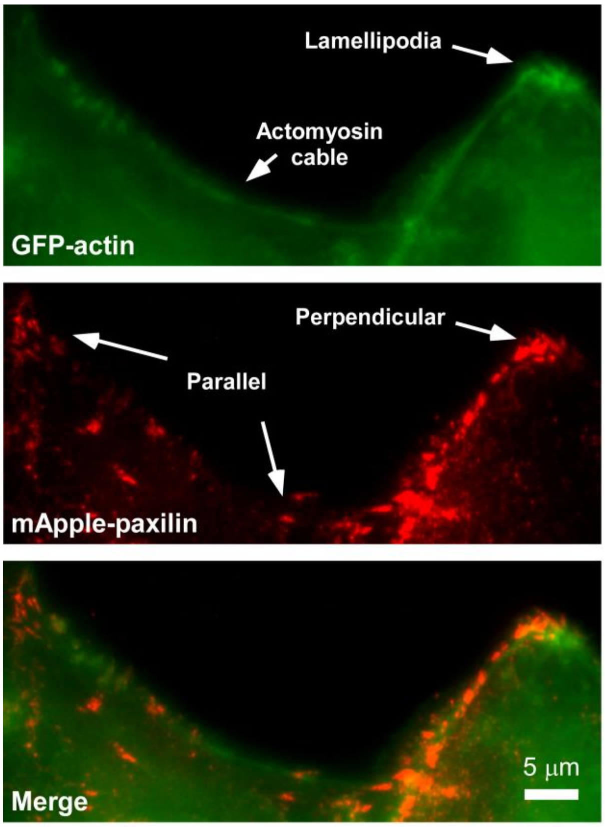

Supplementary Fig. 5 Ravasio et al.

Suppl. Fig. 5 – Live cell imaging of Focal Adhesions

GFP-actin (green, top panel) MDCK cells were transfected with mApple paxilin (red, middle panel). Localization and orientation of focal adhesions confirmed the immunofluorescence data. GFP-actin was visualized by brightfield fluorescence and paxilin adhesion to the substrate by TIRF.

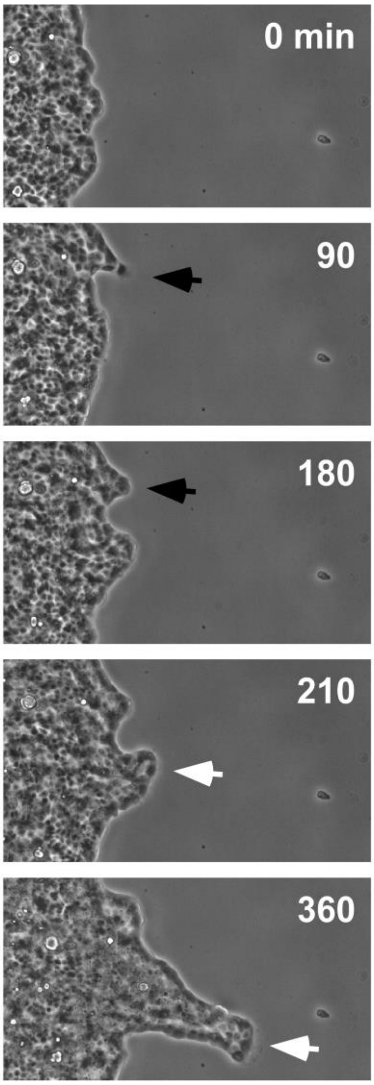

Supplementary Fig. 6 Ravasio et al.

Suppl. Fig. 6 – Migration of MDCK in a model wound experiment

Tug-of-war between crawling and purse-string mechanism can be observed in model wound experiment. After removal of a PDMS stencil, a confluent MDCK tissue started migrating. A number of protrusions immediately appeared (see Suppl. Movie 12). However, the large majority of them stopped their motion after initiating a positive curvature (black arrow). In few cases, the cells at the tip of this positive protrusion were effectively able to sustain their motion over a long period of time (white arrow). In this second case, cells at the tip showed larger lamellipodia and signs of mesenchymal transformation. Substrate was coated with 20 µg/ml fibronectin.
